# Exploring women’s knowledge of abortion legality and association with source of abortion care using population-based survey data in Côte d’Ivoire and Ghana

**DOI:** 10.1186/s12978-024-01871-5

**Published:** 2024-09-12

**Authors:** Grace Sheehy, Caroline Moreau, Easmon Otupiri, Sarah Keogh, Georges Guiella, Suzanne O. Bell

**Affiliations:** 1 Johns Hopkins Bloomberg School of Public Health, Baltimore, MD USA; 2 Kwame Nkrumah University of Science and Technology, Kumasi, Ghana; 3 Guttmacher Institute, New York, NY USA; 4 Institut Supérieur Des Sciences de La Population (ISSP), Université of Ouagadougou, Ouagadougou, Burkina Faso

**Keywords:** Abortion law, Legal knowledge, Ghana, Cote d’Ivoire

## Abstract

**Background:**

Knowledge of the conditions under which abortion is legal is important so that people can advocate for their right to abortion care. Yet minimal research has explored the association between women’s knowledge of abortion legality and the induced abortion care they receive, particularly using population-based survey data.

**Methods:**

Using national survey data collected by Performance Monitoring for Action (PMA) in Côte d’Ivoire and Ghana, we aimed to compare the prevalence of accurate knowledge of abortion legality, factors associated with knowledge of the law, and the association between knowledge of abortion legality and the source of women’s induced abortion care in these two settings. We ran bivariate and multivariable logistic regressions to assess the relationships of interest.

**Results:**

We found that awareness and knowledge of the abortion law were low in both Côte d’Ivoire and Ghana. In Cote d’Ivoire, women who were older, more educated, and with past abortion experience were more likely to be aware of the law. In Ghana, knowledge of the law did not vary significantly by demographic characteristics. However, in Ghana, knowledge of the law was correlated with women’s use of the formal healthcare system for abortion care, with greater use of clinical sources in rural areas.

**Conclusions:**

It is possible that for populations with reduced access to abortion services, such as those in rural areas, knowledge of the law is advantageous for those seeking facility-based abortion care, particularly in settings where abortion is legal for a range of indications. Interventions seeking to improve access to facility-based abortion care in settings where abortion is legal on various grounds should incorporate education on the legal grounds for abortion.

**Supplementary Information:**

The online version contains supplementary material available at 10.1186/s12978-024-01871-5.

## Plain language summary

Knowledge of the conditions under which abortion is legal is important for people to advocate for their right to abortion care, yet minimal research has explored the association between women’s knowledge of abortion legality and the abortion care they receive, particularly using population-based survey data. Using national survey data collected by Performance Monitoring for Action (PMA) in Côte d’Ivoire and Ghana, we aimed to compare the prevalence of accurate knowledge of abortion legality, factors associated with knowledge of the law, and the association between knowledge of abortion legality and the source of women’s abortion care in these two settings. We ran bivariate and multivariable logistic regressions to assess the relationships of interest. We found that awareness and knowledge of the abortion law were low in both Côte d’Ivoire and Ghana. In Côte d’Ivoire, knowledge was unequally distributed according to sociodemographic factors, while such inequities did not exist in Ghana. However, in Ghana, knowledge of the law was correlated with women’s use of the formal healthcare system for abortion care, with greater use of clinical sources in rural areas. It is possible that for populations with reduced access to abortion services, such as those in rural areas, knowledge of the law is advantageous for those seeking facility-based abortion care, particularly in settings where abortion is legal for a range of indications. Interventions seeking to improve access to facility-based abortion care in settings where abortion is legal on various grounds should incorporate education on the legal grounds for abortion.

**Supplementary Information:**

The online version contains supplementary material available at 10.1186/s12978-024-01871-5.

## Introduction

Globally, 41% of reproductive-aged women live in countries with restrictive abortion laws that criminalize abortion in all but a narrow set of circumstances, such as medical grounds (e.g., if the pregnancy poses a threat to life or health) or due to the circumstances of conception (e.g., rape or incest) [1]. The interpretation of these legal grounds varies within and between countries, owing in part to vagueness in statutory language, conflicting guidance between laws, policies, and clinical guidelines, and the arbitrary, inconsistent ways these laws are enforced [2]. These interpretations are exacerbated by anti-abortion stigma and fear of criminal repercussions, both of which can impede access to accurate information about abortion.

Knowledge of the abortion law may directly influence the likelihood of utilizing facility-based abortion services. Qualitative research in Zambia found that women who did not know abortion was a legal option were more likely to use multiple, often increasingly dangerous, abortion methods over the course of their abortion trajectory [3]. In Nepal, less than half (44%) of women seeking post-abortion care knew that abortion was legal, and those who did not know the law were more likely to have received induced abortion care from informal sector providers [4]. A maternal health survey in Ghana conducted in 2017 found that women who reported having an abortion were 2.5 times more likely to have had an unsafe abortion if they did not know the legal status of abortion compared to women who knew the law [5] while another study from Ghana surveyed women seeking treatment for abortion-related complications and found just 8% were aware of the abortion law [6].

It is well supported in the literature that poor, rural, and less educated women have fewer resources to bypass legal restrictions and are significantly more likely to have unsafe abortions than their counterparts [7–9]; for these populations, knowledge of the circumstances under which abortion is legal may be particularly important for accessing facility-based abortion care. A 2016 systematic review found that women often lacked knowledge of the abortion law, with disparities in knowledge by geography, wealth, and education [10]. In the 2017 Ghana Maternal Health Survey, only 10.6% of women were aware abortion was legal for numerous indications [11]. Women in urban parts of the country, with secondary education or higher, and in the highest wealth quintile were more likely to have correct knowledge of the abortion law in the 2008 Demographic and Health Survey [12]. In Ethiopia, knowledge of all legal indications for abortion was significantly lower in rural than in urban rural parts of the country [13].

In this study, we explore knowledge of abortion legality and its connection to induced abortion care seeking in two West African settings that provide a stark contrast in abortion legislation, from highly restrictive in Côte d’Ivoire, where abortion is legal only to save a woman’s life and in cases of rape [14, 15], to more liberal in Ghana, where abortion has been legal for a range of indications since 1985, including in cases of rape, incest, threats to a woman’s health or life, in cases where a woman is mentally incapacitated, and for fetal anomalies [16]. Despite the differing legal environments, in both countries a considerable proportion of abortions are considered unsafe: in Côte d’Ivoire, 62.4% of abortions were classified as least safe (using non-recommended methods by untrained providers) according to a nationally representative 2018 survey [8, 17] and in Ghana, 71% of abortions took place outside of the formal health care system and without a clinical provider in 2017 [18]. In both countries, abortion is legal only in clinical settings.

Using two national surveys in Côte d’Ivoire and Ghana, we aimed to compare the prevalence of accurate knowledge of abortion legality, factors associated with knowledge of the law, and the association between knowledge of abortion legality and the source of women’s induced abortion care in these legally contrasting settings. We hypothesized a stronger relationship between knowledge and facility-based abortion source in Ghana compared to Côte d’Ivoire, given greater available legal indications for abortion in Ghana. We also explored how these associations differed by area of residence (urban versus rural), given disparities in access to healthcare resources between these settings.

## Methods

### Data source and sample

This analysis draws from nationally representative female surveys from Côte d’Ivoire and Ghana collected via Performance Monitoring for Action (PMA). The studies were approved by ethical review boards in both countries (the Comité d'Éthique de la Recherche of Côte d’Ivoire and the Kwame Nkrumah University of Science and Technology (KNUST) Committee on Human Research, Publication and Ethics), the Johns Hopkins Bloomberg School of Public Health Institutional Review Board (IRB), and the Guttmacher Institute’s IRB.

In both countries, a two-stage stratified cluster sampling strategy was used to produce nationally representative samples of reproductive aged women (ages 15–49) [19], including 2,738 women (98.1% response rate) in Côte d’Ivoire and 4,722 women (99.3% response rate) in Ghana. The sampling strategy for this survey is further detailed in prior publications [8, 18, 19]. Our final analytic samples included 2,735 women in Côte d’Ivoire and 4,594 women in Ghana who had responded to all survey items related to knowledge of the abortion law.

### Data collection

The PMA female surveys were conducted by trained resident interviewers in French or a local language in Côte d’Ivoire, and in English or a local language in Ghana. Data were collected in July and August 2018 in Côte d’Ivoire and May and June 2018 in Ghana. The 30-min questionnaire solicited information on a range of topics, including sociodemographic characteristics, pregnancy history, contraceptive use, and abortion knowledge and experiences.

### Measures

Key variables considered in this analysis included (1) awareness and knowledge of the abortion law, (2) source of abortion care, and (3) sociodemographic factors.

*Awareness and knowledge of the abortion law:* Awareness and knowledge were considered as distinct categories, as conceptualized by Assifi et al. in their systematic review on women’s knowledge of abortion laws [10]. Specifically, *awareness* of the law referred to “an individual being aware of a law i.e., knowing that an abortion law exists in a particular country” while knowledge involved identifying “the conditions under which abortion is legal” [10]. We adapted these definitions to align with our survey questions by grouping responses into three categories: not aware, aware, and knowledgeable. In Côte d’Ivoire, women were asked, “Is there a law on abortion in Côte d’Ivoire?” followed by “Are there instances when it is legal to have an abortion in Côte d’Ivoire?”, if they responded positively. In Ghana, women were asked “Are there any circumstances under which the law in Ghana allows a woman to have an abortion?” followed by “Does the law allow a woman to end her pregnancy in the following situations?” Using this information, we defined a three-category variable in each country. In Cote d’Ivoire, the three categories were: (1) not aware of existence of the law (responded “no” to the first question), (2) aware of existence of the law (responded “yes” to the first question, but “no” to the second question), and (3) aware of abortion legality (responded “yes” to this first question and responded “yes” that there are instances when it is legal to have an abortion in Côte d’Ivoire). In Ghana, the three categories were: (1) not aware of abortion legality (responded “no” to the first question), (2) aware of abortion legality (responded “yes” to the first question, but knew only some legal conditions), and (3) knowledgeable about the law (said “yes” to all indications for which abortion is legal).

*Abortion reporting:* PMA explores abortion experiences using two terminologies to improve reporting on induced abortion: respondents were asked whether they had ever done something to remove a pregnancy, and whether they had ever done something to bring back a period when they were worried they were pregnant. Respondents who replied “yes” to either were classified as having had a likely abortion. In Côte d’Ivoire, 216 women reported regulating a period and 497 reported removing a pregnancy, while in Ghana, 564 women reported regulating a period and 730 reported removing a pregnancy.

*Source of abortion care:* Women who reported a likely abortion were asked about the timing, method, and source of abortion care. We categorized a likely abortion source as “clinical” if it took place in a public or private facility, while non-clinical sources included pharmacies, chemist shops, and other sources [20].

*Covariates* included a range of sociodemographic characteristics: age (15–19, 20–29, 30–39, 40–49), marital status (married/cohabitating, divorced/widowed, never married), residence (urban/rural), highest level of education (none, primary, secondary, tertiary), household wealth tertile (high, middle, low), parity, and contraceptive use (a binary measure of whether or not they are a current/recent user, meaning they used contraception in the past 12 months). In Côte d’Ivoire, we also included a measure of perceived abortion stigma [21, 22] via the question “a woman who removes a pregnancy brings shame to her family”, with 5-category Likert scale response options ranging from strongly agree to strongly disagree. We dichotomized response options into agree/disagree, grouping “neither agree nor disagree” into the disagree category. Women in Ghana were not asked this question.

### Analysis

We ran bivariate logistic regressions to explore the unadjusted relationships between (1) awareness/knowledge of the law with sociodemographic characteristics and abortion-related measures among all women, and (2) awareness/knowledge of the law and abortion source (clinical/not clinical) among only women who reported having had a likely abortion. We examined correlations between covariates and excluded some variables with high levels of correlation (> 0.6) from multivariate analyses, including parity and wealth. Next, we ran multivariable multinomial logistic regression models to assess the associations between women’s sociodemographic characteristics and reproductive history, and the three-category measure of awareness/knowledge in each country, with the second category, aware of the law, as the reference category.

Finally, we ran multivariable logistic regression models to assess the adjusted associations between awareness/knowledge of the abortion law and the odds of using a clinical abortion source among women who reported having had an abortion. In both countries, covariates included age, marital status, highest level of education, wealth, and in Côte d’Ivoire, abortion attitudes. We stratified the analysis by urban/rural residence to explore how the association between knowledge of the law and clinical source of abortion care differed by location of residence in each country. We have also included a non-stratified analysis in Appendix 1.

All analyses were conducted using Stata 16.1 (Statcorp LP, College Station, TX) and were weighted to account for unequal probabilities of selection.

## Results

### Sample characteristics

Table 1 presents the sample characteristics for both Côte d’Ivoire and Ghana. In both settings, about two-thirds of women were married (64.9% and 65.4%, respectively) and residing in urban areas (61.4% in Côte d’Ivoire and 51.1% in Ghana). In Côte d’Ivoire, 45.2% had no formal education, while in Ghana, most women had a secondary education (38.3%) or tertiary education (26.8%). While one-quarter of women in Côte d’Ivoire had no children, just 6.7% in Ghana had no children. In Ghana, most women identified as Christian (87%), while in Côte d’Ivoire, 39.5% were Muslim and 35.7% were Catholic or Evangelical. In both countries, most women were not current or recent contraceptive users (70.4% in Côte d’Ivoire and 60.4% in Ghana).

**Table 1:** Sample characteristics of respondents in Côte d’Ivoire and Ghana, 2018^a^

Characteristic	Côte d’Ivoire		Characteristic	Ghana	
	%	N		%	N
Age			Age		
15–19	19.95	540	15–19	16.57	779
20–29	36.09	995	20–29	32.87	1552
30–39	29.09	787	30–39	31.88	1410
40–49	14.87	413	40–49	18.67	853
Marital status			Marital status		
Married/cohabitating	64.92	1767	Married/cohabitating	65.40	2990
Divorced/widowed	4.36	126	Divorced/widowed	10.99	508
Never married	30.72	842	Never married	23.62	1088
Residence			Residence		
Rural	38.57	1062	Rural	48.93	1948
Urban	61.43	1673	Urban	51.07	2646
Highest level of education			Highest level of education		
None	45.23	1254	None	16.27	752
Primary	25.89	714	Primary	18.67	870
Secondary	22.88	613	Secondary	38.26	1796
Tertiary	6.00	152	Tertiary	26.80	1176
Wealth			Wealth		
Lowest/lower quintiles	40.10	1066	Lowest/lower quintiles	41.55	1770
Middle quintile	17.16	517	Middle quintile	18.62	962
Higher/highest quintiles	42.73	1152	Higher/highest quintiles	39.83	1862
Parity			Parity		
0	25.71	703	0	6.73	235
1–2	32.20	866	1–2	40.33	1391
3–4	21.52	590	3–4	30.52	1076
5 +	20.58	572	5 +	22.42	767
Religion			Religion		
Muslim	39.50	1147	Any Christian	87.00	618
Catholic	20.33	544	Muslim	6.27	60
Evangelical	15.39	405	Traditional/Other	4.47	29
Other	13.72	382	No religion	2.27	17
No religion	11.07	257			
Ethnicity			Ethnicity		
Akan	34.67	889	Akan	57.81	401
Mande	20.71	574	Ewe	17.16	124
Gur	14.33	403	Other	25.03	199
Other Ivorian	9.26	273			
Other non-Ivorian	21.03	594			
Current or recent contraceptive user			Current or recent contraceptive user		
No	70.35	1928	No	60.42	2604
Yes	29.65	807	Yes	39.58	1618
Total^b^	100.00	2735		100.00	4594

^a^Percentages are weighted for complex survey design, numbers are unweighted

^b^Some measures have different Ns due to skip patterns and missingness

### Abortion-related measures and awareness of abortion legality

Abortion-specific measures are displayed in Table 2. In both countries, approximately one-quarter of respondents (23.8% and 25.0%) reported they had ever had a likely abortion. Just over one-third of women in both countries (35.1% and 36.3%) who reported having had an abortion received abortion care from a clinical source.

**Table 2:** Percentage distribution of abortion experiences and knowledge in Côte d’Ivoire and Ghana, 2018^a^

Abortion-related measures	Côte d’Ivoire			Ghana	
	%	N	Abortion-related measures	%	N
Ever had a likely abortion^b^			Ever had an abortion^b^		
No	76.24	2087	No	75.01	3463
Yes	23.76	647	Yes	24.99	1131
Clinical source of abortion care^c^			Clinical source of abortion care^c^		
No	64.94	427	No	63.68	124
Yes	35.06	220	Yes	36.32	66
Believes abortion brings shame to woman's family			n/a		
No	42.79	1197			
Yes	57.21	1538			
Knowledge of abortion legality	%	#	Knowledge of abortion legality	%	#
Is there a law on abortion in Côte d’Ivoire?			Are there any circumstances under which the law in Ghana allows a woman to have an abortion?		
Yes	24.27	670	Yes	36.87	1663
No	72.30	1991	No	59.65	2715
Don't know	3.44	74	Don't know	3.48	215
Are there instances when it is legal to have an abortion?^d^			Knowledge of specific legal indications^d^		
Yes	55.40	351	Life at risk	84.52	1397
No	38.49	273	Physical health at risk	54.12	881
Don’t know	6.11	46	Pregnancy is from rape	52.75	883
			Pregnancy is from incest	44.23	718
			Fetal anomaly	52.77	885
			Women is mentally incapacitated	34.20	540
			Women's mental health is at risk	41.19	666
Awareness of abortion legality			Awareness of abortion legality		
Not aware of law	75.73	2065	Not aware of abortion legality	63.14	2930
Aware of law	10.82	319	Aware of abortion legality	31.19	1419
Aware of abortion legality	13.45	351	Knowledgeable about law (i.e., aware of all legal indications)	5.67	242
Total	100.00	2735		100.00	4594

^a^Percentages are weighted for complex survey design, numbers are unweighted

^b^Includes reports of both pregnancy removal and period regulation

^c^In Cote d’Ivoire, n = 647 for abortion source. In Ghana, n = 190 for abortion source, as this question was only asked about abortions in the past 3 years

^d^Only asked to those who said yes to prior question

In Côte d’Ivoire, one-quarter of women (24.3%) were aware of there being a law on abortion, and among them, roughly half (55.4%) were aware that there are legal indications for abortion. In Ghana, over one-third (36.9%) were aware that there are circumstances under which abortion is legal. Among these women, a majority (84.5%) knew abortion was legal when a woman’s life is at risk, and more than half (54.1%) when physical health is at risk or when the pregnancy resulted from rape (52.8%). The legal indications correctly identified by the fewest respondents were incest (44.2%) and mental health (41.2%). Grouping these questions together into our 3-category measure, in Côte d’Ivoire we found that most women (75.7%) were not aware of the abortion law, while 10.8% were aware of the law but did not know of any legal indications, and 13.5% were knowledgeable that there were legal indications. In Ghana, nearly two-thirds (63.1%) were not aware of the law, nearly one-third (31.2%) were aware of the law and some legal indications, and 5.7% were knowledgeable about all legal conditions.

### Factors associated with awareness of abortion legality

Table 3 includes the percentage distribution of the awareness/ knowledge of legality measure according to background characteristics of respondents. In Côte d’Ivoire, awareness/knowledge was significantly associated with age, education, wealth and past abortion experience. Older women, more educated, wealthier women and women who had previously experienced an abortion were more likely to be aware of the law. In Ghana, awareness and knowledge of abortion legality did not vary significantly according to background characteristics. In adjusted models, past abortion, older age, and higher education remained associated with greater awareness of the abortion law in Côte d’Ivoire, while no factors were significantly associated with awareness or knowledge of the law in Ghana (Table 4).

**Table 3:** Percentage distribution of abortion knowledge and respondent characteristics in Côte d’Ivoire and Ghana^a^

Background characteristics	Côte d’Ivoire^b^						Ghana^b^					
	Not aware		Aware of law		Aware of legality		Not aware		Aware		Knowledgeable	
	%	N	%	N	%	N	%	N	%	N	%	N
Age												
15–19	**82.16**	**444**	**9.17**	**49**	**8.67**	**47**	64.54	529	29.91	218	5.54	31
20–29	**75.74**	**744**	**11.46**	**133**	**12.80**	**118**	62.38	960	32.12	507	5.50	84
30–39	**72.09**	**570**	**11.34**	**93**	**16.57**	**124**	64.00	907	30.03	419	5.97	83
40–49	**74.19**	**307**	**10.49**	**44**	**15.31**	**62**	61.78	534	32.65	275	5.56	44
Marital status												
Married/cohabitating	76.76	1358	9.90	190	13.34	219	63.27	1904	31.20	927	5.54	158
Divorced/widowed	78.29	94	8.10	14	13.61	18	64.31	321	28.78	154	6.91	32
Never married	73.19	613	13.17	115	13.64	114	62.55	704	31.95	331	5.49	52
Residence												
Rural	79.72	834	7.98	94	12.30	134	62.58	1251	32.29	619	5.13	78
Urban	73.23	1231	12.61	225	14.17	217	63.68	1679	30.13	800	6.19	164
Highest level of education												
None	**85.87**	**1064**	**6.83**	**102**	**7.30**	**88**	66.05	520	29.95	208	3.99	24
Primary	**71.93**	**523**	**10.36**	**83**	**17.71**	**108**	65.80	587	28.85	240	5.35	43
Secondary	**65.09**	**395**	**17.36**	**106**	**17.55**	**112**	64.98	1188	29.63	527	5.39	80
Tertiary	**55.90**	**81**	**18.17**	**28**	**25.93**	**43**	56.89	635	35.79	444	7.31	95
Parity												
0	76.46	534	12.29	87	11.25	82	58.80	136	37.06	86	4.14	13
1–2	73.08	626	11.76	114	15.16	126	66.57	896	27.71	409	5.72	84
3–4	77.29	460	9.59	61	13.12	69	63.77	694	30.42	324	5.81	58
5 +	77.44	443	8.77	56	13.79	73	60.87	491	33.18	241	5.94	35
Wealth												
Low	**81.31**	**860**	**6.93**	**83**	**11.77**	**123**	61.26	1144	33.42	560	5.32	65
Middle	**79.04**	**407**	**10.71**	**63**	**10.26**	**47**	66.16	631	29.07	288	4.77	43
High	**69.17**	**798**	**14.53**	**173**	**16.30**	**181**	63.69	1155	29.85	571	6.45	134
Current or recent family planning user												
No	**79.28**	**1524**	**9.33**	**192**	**11.38**	**212**	64.15	1702	30.80	782	5.05	119
Yes	**67.31**	**541**	**14.36**	**127**	**18.33**	**139**	61.54	986	31.69	525	6.77	107
Ever had an abortion												
No	**78.84**	**1657**	**9.32**	**214**	**11.84**	**216**	62.65	2227	31.75	1063	5.60	170
Yes	**65.73**	**407**	**15.67**	**105**	**18.60**	**135**	64.63	703	29.51	356	5.86	72
Believes abortion brings shame to woman's family												
No	72.47	876	11.69	151	15.85	170	n/a	n/a	n/a	n/a	n/a	n/a
Yes	78.17	1189	10.18	168	11.65	181	n/a	n/a	n/a	n/a	n/a	n/a
Abortion method used^c^												
Surgery	63.59	123	20.02	38	16.39	38	66.70	21	24.77	13	8.52	4
Mifepristone/Misoprostol	62.25	19	21.71	6	16.04	5	77.51	39	18.46	15	4.03	4
Other pills	64.13	69	13.64	15	22.23	28	59.23	59	36.20	37	4.58	4
Traditional	68.21	195	12.52	45	19.27	64	62.79	160	31.99	87	5.23	13
Used clinical source for abortion care^c^												
No	66.70	271	13.76	64	19.54	92	71.09	81	22.85	34	6.06	9
Yes	63.95	136	19.20	41	16.86	43	64.38	38	29.43	24	6.20	4
Abortion was least safe^c^												
No	63.12	144	19.67	46	17.21	47	68.60	83	25.19	42	6.22	10
Yes	67.40	262	13.09	58	19.52	88	67.85	36	26.43	17	5.72	3

^a^Percentages are weighted for complex survey design, numbers are unweighted

^b^Bolded estimates are significant at p < 0.05

^c^In Cote d’Ivoire, n = 647 for abortion method, source and safety. In Ghana, n = 200 for abortion method and n = 190 for abortion source, as these questions were only asked about abortions in the past 3 years

**Table 4:** Factors associated with awareness of the law in Côte d’Ivoire and Ghana, adjusted models^a^

	Cote d’Ivoire^b^				Ghana^c^			
	Not aware of the law		Aware of abortion legality		Not aware of the law		Knowledgeable about law	
	aRRR	95% CI	aRRR	95% CI	aRRR	95% CI	aRRR	95% CI
Abortion history								
Ever had a likely abortion	**0.66**	**(0.47–0.94)**	0.82	(0.47**–**1.42)	1.16	(0.84**–**1.62)	1.02	(0.57**–**1.81)
Age								
15–19	Ref	–	Ref	–	Ref	–	Ref	–
20–29	**0.60**	**(0.39–0.92)**	1.08	(0.58**–**2.01)	0.85	(0.56**–**1.29)	0.64	(0.34**–**1.22)
30–39	**0.50**	**(0.29–0.85)**	1.42	(0.75**–**2.71)	0.84	(0.53**–**1.33)	0.74	(0.34**–**1.58)
40–49	**0.48**	**(0.26–0.87)**	1.37	(0.65**–**2.89)	0.72	(0.43**–**1.21)	0.67	(0.29**–**1.54)
Marital status								
Currently married/cohabitating	Ref	–	Ref	–	Ref	–	Ref	–
Divorced/widowed	1.31	(0.64**–**2.66)	1.22	(0.57**–**2.59)	1.13	(0.81**–**1.57)	1.34	(0.74**–**2.45)
Never married	0.84	(0.59**–**1.20)	0.89	(0.55**–**1.44)	0.95	(0.61**–**1.46)	0.71	(0.31**–**1.61)
Residence								
Rural	Ref	–	Ref	–	Ref	–	Ref	–
Urban	0.78	(0.46**–**1.32)	0.72	(0.36**–**1.47)	1.21	(0.78**–**1.89)	1.29	(0.31**–**5.34)
Highest level of education								
None	Ref	–	Ref	–	Ref	–	Ref	–
Primary	**0.60**	**(0.43–0.83)**	1.70	(0.98**–**2.97)	1.00	(0.66**–**1.49)	1.39	(0.71**–**2.74)
Secondary	**0.30**	**(0.19–0.48)**	1.19	(0.72**–**1.97)	0.86	(0.53**–**1.40)	1.29	(0.67**–**2.50)
Tertiary	**0.34**	**(0.18–0.64)**	1.59	(0.96**–**2.61)	0.62	(0.37**–**1.07)	1.49	(0.77**–**2.87)
Contraceptive use								
Current or recent contraceptive user	0.75	(0.55**–**1.02)	1.07	(0.73**–**1.58)	0.95	(0.75**–**1.21)	1.29	(0.88**–**1.89)
Abortion attitudes								
Agrees abortion brings shame to woman's family	1.07	(0.73**–**1.56)	0.83	(0.59**–**1.15)	–	–	–	–

^a^Bolded estimates are statistically significant at p < 0.05; aRRR: adjusted relative risk ratio

^b^“Aware of existence of the law” is the reference category in Cote d’Ivoire

^c^“Aware of abortion legality” is the reference category in Ghana

### Relationship between awareness of abortion legality and abortion care received

Finally, we examined the association between awareness/knowledge of the law and the odds of using a clinical abortion source, stratifying by urban/rural residence. In Ghana, adjusting for demographic characteristics, knowledge of the law significantly increased the odds of using a clinical abortion source in rural parts of the country, although our confidence intervals were very wide (aOR: 15.3, 95% CI 3.1–75.3, p < 0.001). In contrast, knowledge of the law decreased the odds of using a clinical abortion source in urban parts of Ghana, although this finding was only borderline statistically significant after adjustment (aOR: 0.26, 95% CI 0.1–1.02, p = 0.053) (Table 5). Similar patterns were found in Côte d’Ivoire, although associations were smaller and not statistically significant (Table 5). In Ghana, older age and having never been married were significantly associated with use of a clinical source in rural areas, but not in urban areas, holding all else constant. In urban areas of Côte d’Ivoire older women were more likely to use a clinical source in our bivariate analysis, although this finding did not remain significant after adjustment, while never married women were less likely than married/cohabitating women to use a clinical source. In rural areas in Cote d’Ivoire, women with primary education were more likely to use a clinical source than those with no education, holding all else constant (Table 5). In sensitivity tests, awareness/knowledge of the law was not significantly associated with use of a clinical abortion source in either country when data was not stratified by urban/rural residence (Appendix 1).

**Table 5:** Adjusted association between awareness of law and the odds of using a clinical abortion source, stratified by rural/urban^a^

	Cote d’Ivoire				Ghana			
	RURAL (n = 247)		URBAN (n = 400)		RURAL (n = 70)		URBAN (n = 114)	
	aOR	95% CI	aOR	95% CI	aOR	95% CI	aOR	95% CI
Awareness of the law								
Not aware	Ref	–	Ref		Ref	–	Ref	–
Aware of law	1.59	(0.45–5.59)	1.03	(0.55–1.93)	3.76	(0.96–14.73)	1.06	(0.40–2.82)
Aware of legality	1.09	(0.44–2.68)	0.65	(0.35–1.21)	**15.30**	**(3.11**–**75.30)**	0.26	(0.07–1.02)
Age								
15–19	Ref	–	Ref		Ref	–	Ref	–
20–29	2.05	(0.47–9.03)	1.73	(0.65–4.61)	**7.89**	**(1.10**–**56.58)**	0.87	(0.19–4.05)
30–39	2.55	(0.48–13.59)	2.48	(0.83–7.40)	**20.51**	**(2.16**–**194.49)**	3.05	(0.73–12.78)
40–49	2.66	(0.59–12.01)	1.84	(0.51–6.61)	**11.67**	**(1.29**–**105.48)**	3.20	(0.14–71.67)
Marital status								
Currently married/cohabitating	Ref	–	Ref		Ref	–	Ref	–
Divorced/widowed	0.91	(0.32–2.63)	0.59	(0.21–1.69)	0.58	(0.16–2.14)	1.09	(0.14–8.77)
Never married	0.92	(0.27–3.08)	**0.57**	**(0.33**–**0.97)**	**7.06**	**(1.20**–**41.56)**	2.23	(0.44–11.40)
Highest level of education								
None	Ref	–	Ref		Ref	–	Ref	–
Primary	**2.48**	(1.11–5.50)	1.13	(0.66–1.93)	1.82	(0.14–23.89)	1.28	(0.12–13.13)
Secondary	1.44	(0.39–5.33)	1.67	(0.90–3.11)	2.35	(0.39–13.95)	0.59	(0.11–3.23)
Tertiary	1.00	–	1.39	(0.58–3.32)	0.94	(0.10–9.27)	0.71	(0.17–2.99)
Wealth								
Low	Ref	–	Ref		Ref	–	Ref	–
Middle	1.13	(0.42–2.99)	1.28	(0.25–6.65)	0.92	(0.27–3.10)	0.78	(0.14–4.30)
High	2.35	(0.59–9.30)	2.20	(0.58–8.41)	0.56	(0.04–8.40)	1.05	(0.36–3.02)
Abortion attitudes								
Agrees abortion brings shame to woman's family	0.49	(0.21–1.13)	0.90	(0.57–1.42)	–	–	–	–

^a^Bolded estimates are statistically significant at p < 0.05

*aOR* Adjusted Odds Ratio, *CI* Confidence Interval

## Discussion

Minimal research has explored the association between women’s knowledge of abortion legality and the abortion care they receive, particularly using population-based survey data. Across two countries with diverse legal conditions for abortion in West Africa, we found awareness and knowledge of the law were low. More women were aware of the law (31.2%) than were knowledgeable about the specifics of the law (5.7%), a gap that is consistently reported in low- and middle-income countries with more liberal abortion laws, such as Ethiopia, where 27% of women were aware of the law but only 5% had complete knowledge [13]. In Côte d’Ivoire, women who were older, more educated, and with past likely abortion experience were more likely to be aware of the law, while in Ghana, knowledge of the law did not vary significantly by demographic characteristics.

We also assessed the relationship between knowledge of the law and use of a clinical source of abortion care. We did not find a relationship in Cote d’Ivoire, while in Ghana, greater knowledge was associated with increased odds of having an abortion in a clinical setting in rural parts of the country, holding all else constant. Differences by area of residence could reflect broader inequities in access to care that intersect with women’s awareness of the legal barriers to access abortion in a country which provides limited safe abortion services. However, our stratified sample was small and our confidence intervals were wide, which could have contributed to this finding in rural Ghana, and our analysis does not conclusively show that knowledge of the law is associated with greater use of a clinical abortion source.

Past research in Ghana has found a significant association between knowing the legal status of abortion and the odds of having a safe abortion [5]. We provide further insights into differential effects of legal knowledge and access to abortion care, showing the connection could potentially be more relevant in settings where abortion is legal for various indications (such as Ghana) than in more restrictive settings (such as Cote d’Ivoire), where social and economic factors may be more influential than legal knowledge in accessing facility-based abortion care; however, further research is needed to determine whether this association is relevant in other settings. Additionally, we examined use of facility-based abortion care, which is not inherently synonymous with safe abortion care; unsafe abortion care can be provided in health facilities and abortions can also be managed safely outside facilities, in line with WHO recommendations [23]. However, we were interested in exploring whether knowing the abortion law had an association with women’s likelihood of seeking care in the formal health sector specifically, rather than their choice of method or a composite measure of abortion safety.

In legally restrictive settings like Cote d’Ivoire, expanding abortion access outside formal channels (i.e., via community-based distribution of medication abortion) may be a more effective approach for reducing the harms from unsafe abortion and potentially improving access to safe and/or facility-based abortion care than expanding knowledge of the law. In contrast, in countries where abortion is legal for a broader range of indications, including mental health, expanding knowledge of the abortion law could be a useful intervention for increasing access to safe and/or facility-based abortion care. In these settings, there is more opportunity for communities and providers to interpret the law broadly so that abortion services can be made more widely available, and knowledge of legal indications such as mental health could be beneficial for people seeking facility-based abortion care. This broad interpretation of the law may be especially impactful for populations with reduced access to abortion services, such as those in rural areas. Future research should further explore rural–urban dynamics in abortion knowledge and access.

While knowledge of the law is potentially valuable for ensuring abortion-seekers can advocate for their right to care, most abortions are sought for social and economic reasons, rather than common legal indications such as threat to life or health or instances of rape [24]. Thus, even complete knowledge of these indications is inadequate for expanding access to safe abortion care for the majority of abortion care seekers; in settings where abortion is legally restricted, such as Cote d’Ivoire, knowledge of the law may be irrelevant to access to clinical care because most people are not accessing abortion care for reasons that align with narrow legal indications. Alternatively, in these settings, knowledge of the law may be inversely associated with seeking facility-based abortion care, as people who are aware of the legal restrictions may avoid facility settings.

Abortion restrictions lead to preventable morbidity and mortality, which can be avoided by liberalizing abortion laws to allow the legal provision of abortion care without restriction [25, 26]. Consequently, In their 2022 Abortion Care Guideline, the WHO recommends complete decriminalization of abortion, thus removing it from all penal/criminal laws and eliminating all associated criminal penalties, making abortion available on request [23].

### Limitations

Our study is not without limitations. We drew from cross-sectional survey data, and thus cannot determine the temporality of the relationships we measure; specifically, we cannot determine whether knowledge of the law leads to access to clinical abortion sources, or if those who access abortion care in clinical settings learned about the law at the time of (or after) their abortion. Our study does not identify whether there is a causal link between knowledge of the law and use of a clinical abortion source; this is an area where further research is needed. Further, we do not examine the relationship between knowledge of the law and abortion safety, since non-clinical abortion sources can also provide safe abortion care, i.e., via self-managed abortion with medication abortion. Abortion is grossly underreported in surveys, and if reporting is associated with knowledge of the law, this could bias our estimates. Further, the samples for our stratified analyses were small, including only those who reported an abortion, resulting in wide confidence intervals. Finally, there were limitations to our knowledge measures, which impeded our ability to make direct comparisons between the two countries. In Côte d’Ivoire, women were not asked the specific conditions under which abortion is legal, and thus in this setting, we were only able to measure awareness of the law.

## Conclusion

Overall, we found awareness and knowledge of the law were low among women in both Côte d’Ivoire and Ghana. In Côte d’Ivoire, women who were older, more educated, and with past likely abortion experience were more likely to be aware of the law, while in Ghana, knowledge of the law did not vary significantly by demographic characteristics. In rural Ghana, knowledge of the law was associated with increased likelihood of using a clinical abortion source, while there was no association with use of a clinical source in Cote d’Ivoire. Our findings do not show a strong association between legal knowledge and use of a clinical abortion source in these two settings; however further research on the role of legal knowledge in abortion care-seeking is needed.

## Supplementary Information

Supplementary material 1.

## Data Availability

Data from Cote d’Ivoire for this study are publicly available and can be requested online at pmadata.org. De-identified data from Ghana are available from the Guttmacher Institute upon reasonable request to researchers who wish to use the data for scholarly analysis. To discuss obtaining copies of these datasets, please contact popcenter@guttmacher.org with the detailed protocol for your proposed study, and information about the funding and resources you have to carry out the study.
