# Supplementary material for: Exploring women’s knowledge of abortion legality and association with source of abortion care using population-based survey data in Côte d’Ivoire and Ghana
Source: Reprod Health. 2024 Sep 12;21:132. doi: 10.1186/s12978-024-01871-5 (PMC11391610; doi:10.1186/s12978-024-01871-5)
Supplement: Supplementary file 1 — Supplementary material 1. [file 12978_2024_1871_MOESM1_ESM.docx]

**Appendix 1. Sensitivity analysis: Odds of using a clinical abortion source**

This analysis replicates the analysis run for Table 4 (adjusted association between awareness of the law and odds of using a clinical abortion source) without rural/urban stratification.

|  | **COTE D’IVOIRE (n=647)** | | | | **GHANA (n=184)** | | |
| --- | --- | --- | --- | --- | --- | --- | --- |
|  | **aOR** | | **95% CI** | | **aOR** | **95% CI** | |
| **Awareness of the law** |  |  | |  |  |  |  |
| Not aware | Ref | - | |  | Ref | - |  |
| Aware of law | 1.17 | (0.67 to | | 2.05) | 1.56 | (0.82 to | 2.99) |
| Aware of legality | 0.78 | (0.46 to | | 1.32) | 1.26 | (0.35 to | 4.48) |
| **Age** |  |  | |  |  |  |  |
| 15-19 | Ref | - | |  | Ref | - |  |
| 20-29 | 1.66 | (0.73 to | | 3.78) | 1.56 | (0.47 to | 5.14) |
| 30-39 | 2.35 | (0.94 to | | 5.88) | 5.49 | (1.67 to | 18.02) |
| 40-49 | 2.00 | (0.71 to | | 5.60) | 3.36 | (0.61 to | 18.48) |
| **Marital status** |  |  | |  |  |  |  |
| Currently married/ cohabitating | Ref | - | |  | Ref | - |  |
| Divorced/widowed | 0.71 | (0.34 to | | 1.47) | 0.90 | (0.29 to | 2.77) |
| Never married | 0.63 | (0.39 to | | 1.00) | 2.59 | (0.81 to | 8.33) |
| **Highest level of education** |  |  | |  |  |  |  |
| None | Ref | - | |  | Ref | - |  |
| Primary | 1.55 | (0.96 to | | 2.48) | 1.34 | (0.28 to | 6.40) |
| Secondary | 1.70 | (0.96 to | | 3.02) | 1.17 | (0.43 to | 3.18) |
| Tertiary | 1.51 | (0.64 to | | 3.53) | 1.05 | (0.39 to | 2.85) |
| **Wealth** |  |  | |  |  |  |  |
| Low | Ref | - | |  | Ref | - |  |
| Middle | 1.29 | (0.58 to | | 2.88) | 0.70 | (0.29 to | 1.69) |
| High | 2.20 | (0.98 to | | 4.96) | 0.74 | (0.26 to | 2.13) |
| **Residence** |  |  | |  |  |  |  |
| Rural | Ref | - | |  | Ref | - |  |
| Urban | 1.22 | (0.58 to | | 2.58) | 0.84 | (0.30 to | 2.38) |
| **Abortion attitudes** |  |  | |  |  |  |  |
| Agrees abortion brings shame to woman's family | 0.74 | (0.49 to | | 1.10) | - | - | - |
